# Investigating the Effective Performance of Sandwich Panel with Petal Star-Triangular Core Using VAM-Based Equivalent Model

**DOI:** 10.3390/ma15186407

**Published:** 2022-09-15

**Authors:** Xinlong Yang, Zhen Wang, Yifeng Zhong, Rong Liu

**Affiliations:** 1 School of Civil Engineering, Chongqing University, Chongqing 400045, China; 2 Key Laboratory of New Technology for Construction of Cities in Mountain Area, Chongqing University, Chongqing 400045, China

**Keywords:** honeycomb sandwich panel, negative Poisson’s ratio, static and dynamic performance, variational asymptotic method

## Abstract

On the basis of star-shaped core sandwich panel, a novel sandwich panel with petal-triangle core (SP-PSC) was proposed to improve the negative Poisson’s ratio (NPR) effect while retaining the characteristics of light weight and high strength. To study the complex structure more conveniently and quickly, a variational asymptotic method-based equivalent two-dimensional model (2D-EPM) was developed. The accuracy and efficiency of 2D-EPM were verified by the three-point bending experiment data and the 3D FE model results under different boundary and load conditions. The effects of the geometric parameters on the equivalent stiffness, buckling, natural frequency and NPR effect were also investigated. To increase the NPR of SP-PSC, the material of facesheet was changed from isotropic material to unidirectional CFPR material, and the influence of the material anisotropy on the NPR effect of SP-PSC was investigated. It is found that the NPR of SP-PSC increased first and then decreased with the increase in the fiber angle, reaching the maximum value at 40–$50^{\circ }$. At the same time, this law is applicable to SP-PSC with different material or geometric parameters. Finally, two improved cores, petal star-triangular core with X-shaped ligaments (PSC-X) and double-arc star-shaped core (DSC), were proposed and compared with SP-PSC in equivalent stiffness and recovered local fields to demonstrate their advantages. Compared with the original plate, the stress concentration and equivalent stiffness of the two improved PSCs significantly improved.

## 1. Introduction

Since the beginning of the 21st century, metamaterials have gradually developed into an important branch of new material technology. Metamaterial refers to the design of the internal structure of materials, so as to artificially control various properties to obtain new materials that are not available in nature. The negative Poisson’s ratio (NPR) material is a typical metamaterial, and its Poisson’s ratio characteristics are against intuition. The NPR material was first proposed by Lakes [1] in 1987 and then attracted a large number of interests. After decades of development, NPR materials have been vigorously developed in many fields. For example, the NPR material is applied to the anti-collision device of the vehicle to achieve the effect of energy absorption and cushioning.

Auxetic materials, namely negative Poisson’s ratio (NPR) materials, have many novel and excellent properties which can be enhanced by virtue of their NPR effect. Firstly, auxetic materials have stronger hardness or indentation resistance than non-auxetic materials because the auxetic material contracts laterally and flows into the immediate region of impact, which leads to an increase in density and affects indentation resistance for auxetic materials, but non-auxetic materials flow laterally away from the region of impact during an impact. In addition, according to classical elasticity theory, the hardness (H) of auxetic materials decreased with the enhancement of negative Poisson’s ratio ($H\propto {\left(\frac{E}{1-\mu^{2}}\right)}^{\gamma },\gamma$ is sensitivity index [2]). Secondly, the NPR material has a better shear modulus. It can be seen from $G=\frac{E}{2(1+\mu )}$ (*E* is young’s modulus, *G* is shear modulus, and μ is Poisson’s ratio) that the shear modulus *G* increases as μ approaches −1 [3]. Choi and Lakes [4] demonstrated an improvement in fracture toughness of re-entrant auxetic foams. Therefore, it is valuable to study the NPR of SP-PSC, especially to find a method that can improve the NPR effect.

The NPR materials can be grouped into three categories: re-entrant [5], chiral [6] and rotating rigid structures [7]. The traditional honeycomb re-entrant structure and its auxetic behavior were first proposed by Gibon et al. [5] and Master and Evans [8]. Some re-entrant profiles, such as the star structure presented by Theocaris et al. [9], even have a NPR effect in both directions. The main feature of a re-entrant structure is the concave. The core layer of the sandwich panel studied in this article is a new annular concave structure based on the star structure. In addition, not only can the in-plane re-entrant form an NPR structure, but the spatial structure can also become a re-entrant structure as long as the concave is arranged properly, such as the elastic porous solid studied by Lakes et al. [10]. The comprehensive research on the spatial re-entrant structure is also known as “bucklicystals”.

The honeycomb sandwich panel has attracted much attention as a popular structure. Geramizadeh et al. [11] proved that the facesheet thickness had a great impact on the performance of sandwich panels. In fact, the effective performance of honeycomb sandwich panels is quite different due to the different forms of core layers. Geramizadeh et al. [12] found that rounding regular hexagonal structures can effectively improve mechanical properties. The pursuit of a core layer with excellent performance has become the forward direction of the honeycomb sandwich panel. This paper argues that replacing the core layer with the NPR structure, which can better meet some practical requirements, would improve some sandwich panel performance.

The sandwich panel with a petal star-triangular core (short for SP-PSC) studied in this paper evolved from the regular star structure proposed by Theocaris et al. [9], which combines the advantages of the honeycomb sandwich structure and the NPR effect.

Many theories have been put forward to calculate the performance of sandwich panels, such as the Gibson formula [13], energy method [14], the homogenization method [15], and many other theories. To address the issue of deformation authenticity, a series of related theories have been put forward, such as first-order shear theory [16], high-order shear theory [17], layered theory [18], zig-zag theory, etc. In addition to the study of static deformation, dynamics is also important. For the analysis of damping performance, the complex eigenvalue method (CM) and the modal strain energy method (MSE) were derived to solve many dynamic problems [19]. Gohari et al. [20,21] and Wang et al. [22] mainly studied the numerical simulation and analytical solution of different carbon fiber reinforced composites. Although the three-dimensional model of the honeycomb sandwich panel can obtain more accurate results, it was too time-consuming and inefficient to be applied in practice.

Recently, the variational asymptotic method (VAM) was developed to build a rigorous dimensional reduction plate/shell model with a good balance of efficiency and accuracy [23,24,25]. The essence of the variational asymptotic method is to transform the problem of solving the definite solution of complex elasticity into the problem of solving the extreme value of functional. Finally, the problem is summarized as solving the system of linear algebraic equations. The two-dimensional equivalent model obtained by VAM has high accuracy and efficiency, which has been verified by many scholars [26,27,28]. If the three-dimensional model of SP-PSC is equivalent to the two-dimensional model using VAM, the calculation time can be greatly saved, and the efficiency can be very high while ensuring a certain accuracy.

In this study, the three-point bending test for 3D printer samples of sandwich panels with petal-star cores (SP-PSC) was carried out using an electronic universal testing machine. The calculation accuracy and efficiency of 2D-EPM were verified by comparing them with the experimental data and 3D-FEM results under different boundary and load conditions. The effects of geometric parameters (including angles $\theta_{1}$ and $\theta_{2}$, as well as height ratio) on the stiffness, buckling, natural frequency, and NPR of SP-PSC were studied. To improve the NPR effect of SP-PSC, the facesheet material was changed from isotropic materials to unidirectional composites. Finally, based on the mechanism of SP-PSC, two improved cores were proposed, namely PSC-X and DSC. The findings of this study can be used as a guide for parameter optimization of sandwich plates with complicated cores.

## 2. VAM Procedure

### 2.1. Kinematics of SP-PSC

**Figure 1:**
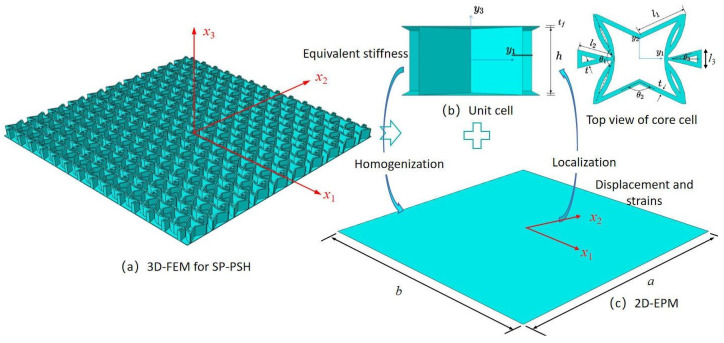
Dimension reduction analysis of the sandwich panel with petal star-triangular core (SP-PSC) using variational asymptotic method (VAM) (the top facesheet is removed for better view).

Two coordinate systems are introduced in multiscale modeling: (1) the global coordinates $x=\left(x_{1},x_{2},x_{3}\right)$ describing the macro plate; (2) the local coordinates $y=\left(y_{1},y_{2},y_{3}\right)$ describing the unit cell, which is parallel to $\left(x_{1},x_{2},x_{3}\right)$. The dimension reduction analysis of the SP-PSC is shown in Figure 1. That is, the three-dimensional FE model (3D-FEM) analysis of SP-PSC is reduced to the constitutive modeling over the typical 3D unit cell and 2D equivalent plate model analysis (2D-EPM). As a result, the original displacement function of SP-PSC can be expressed as a displacement function defined along the reference plane $x_{1}-x_{2}$ ($x_{3}$ disappears), with partial derivative as

(1) $$\frac{\partial u\left(x_{\alpha };y_{i}\right)}{\partial x_{\alpha }}={\left.\frac{\partial u\left(x_{\alpha };y_{i}\right)}{\partial x_{\alpha }}\right|}_{y_{i}=const}+{\left.\frac{1}{\zeta }\frac{\partial u\left(x_{\alpha };y_{i}\right)}{\partial y_{i}}\right|}_{x_{\alpha }=const}\equiv u_{,\alpha }+\frac{1}{\zeta }u_{|i},$$

where ζ denotes the ratio of the micro- and macro-scales.

### 2.2. Step 1: Equivalent 2D Displacements from 3D Displacements

To develop the reduced plate model of SP-PSC using VAM, the 3D displacement field $u_{i}$ of the original SP-PSC is represented by 2D plate variables ${\bar{u}}_{i}$, such that

(2) $$\begin{array}{cc} \; & u_{1}\left(x_{1},x_{2},y_{3}\right)=\underline{{\bar{u}}_{1}\left(x_{1},x_{2}\right)-\zeta y_{3}{\bar{u}}_{3,1}}+w_{1}\left(x_{1},x_{2},y_{3}\right)\\ \; & u_{2}\left(x_{1},x_{2},y_{3}\right)=\underline{{\bar{u}}_{2}\left(x_{1},x_{2}\right)-\zeta y_{3}{\bar{u}}_{3,2}}+w_{2}\left(x_{1},x_{2},y_{3}\right)\\ \; & u_{3}\left(x_{1},x_{2},y_{3}\right)=\underline{{\bar{u}}_{3}\left(x_{1},x_{2}\right)}+w_{3}\left(x_{1},x_{2},y_{3}\right) \end{array},$$

where $w_{i}$ denote unknown warping functions that cannot be considered in the classic plate theory. The underlined terms should meet the constraints as

(3) $${\bar{u}}_{1}=\langle u_{1}\rangle +\langle \zeta y_{3}\rangle {\bar{u}}_{3,1},\;{\bar{u}}_{2}=\langle u_{2}\rangle +\langle \zeta y_{3}\rangle {\bar{u}}_{3,2},\;{\bar{u}}_{3}=\langle u_{3}\rangle ,$$

where 〈·〉 denotes the volume integral over the unit cell.

If the origin of local coordinates is located at the geometric center of the 3D unit cell, the 2-D displacements are the average of the corresponding 3-D displacements, and the warping functions are constrained by

(4) $$\langle \zeta w_{i}\rangle =0.$$

The 3-D strain field is obtained based on the concept of rotation tensor decomposition [29] as

(5) $$\varepsilon_{ij}=\frac{1}{2}\left(\frac{\partial u_{i}}{\partial x_{j}}+\frac{\partial u_{j}}{\partial x_{i}}\right).$$

The change of variables is implemented into the original 3-D warping functions,

(6) $$w_{i}\left(x_{1},x_{2},y_{3}\right)=\zeta y_{3}\varphi_{i}\left(x_{1},x_{2}\right)+v_{i}\left(x_{1},x_{2},y_{3}\right),$$

where $\varphi_{1}$ and $\varphi_{2}$ represent transverse normal rotations about the $x_{2}$ and $x_{1}$ axes, respectively, and $\varphi_{3}$ represents transverse normal elongation along the $x_{3}$ axis.

Substituting Equations (2) and (6) into Equation (5) and ignoring higher-order terms that have little influence on the total energy, we obtain the explicit expression of the 3D strain field as

(7) $$\begin{array}{cc} \; & \varepsilon_{11}=\epsilon_{11}+\zeta y_{3}\kappa_{11}+\zeta y_{3}\varphi_{1,1}+v_{1,1},\\ \; & 2\varepsilon_{12}=2\epsilon_{12}+2\zeta y_{3}\kappa_{12}+\zeta y_{3}\varphi_{2,1}+v_{2,1}+\zeta y_{3}\varphi_{1,2}+v_{1,2},\\ \; & \varepsilon_{22}=\epsilon_{22}+\zeta y_{3}\kappa_{22}+\zeta y_{3}\varphi_{2,2}+v_{2,2},\\ \; & 2\varepsilon_{13}=\varphi_{1}+v_{1,3}+\zeta y_{3}\varphi_{3,1}+v_{3,1},\\ \; & 2\varepsilon_{23}=\varphi_{2}+v_{2,3}+\zeta y_{3}\varphi_{3,2}+v_{3,2},\\ \; & \varepsilon_{33}=\varphi_{3}+v_{3,3}, \end{array}$$

where $\epsilon_{\alpha \beta }$ and $\kappa_{\alpha \beta }$ can be defined as

(8) $$\epsilon_{\alpha \beta }\left(x_{1},x_{2}\right)=\frac{1}{2}\left({\bar{u}}_{\alpha ,\beta }+{\bar{u}}_{\beta ,\alpha }\right),\;\kappa_{\alpha \beta }\left(x_{1},x_{2}\right)=-{\bar{u}}_{3,\alpha \beta }.$$

The 3D strain field E can be expressed in matrix form as

(9) $$\begin{array}{c} E_{e}={\left[\varepsilon_{11}\;\varepsilon_{22}\;2\varepsilon_{12}\right]}^{T}=\epsilon +\zeta y_{3}\kappa +I_{\alpha }\left(\zeta y_{3}\varphi_{∥,\alpha }+v_{∥,\alpha }\right),\\ 2E_{s}={\left[2\varepsilon_{13}\;2\varepsilon_{23}\right]}^{T}=\varphi_{∥}+v_{∥,3}+e_{\alpha }\left(\zeta y_{3}\varphi_{3,\alpha }+v_{3,\alpha }\right),\\ E_{t}=\varepsilon_{33}=\varphi_{3}+v_{3,3}, \end{array}$$

where ${\left(\right)}_{\left|\right|}={\left[{\left(\right)}_{1}\;{\left(\right)}_{2}\right]}^{T}$, $\epsilon ={\left[\begin{array}{ccc} \epsilon_{11} & 2\epsilon_{12} & \epsilon_{22} \end{array}\right]}^{T}$, $\kappa ={\left[\kappa_{11}\;\kappa_{12}+\kappa_{21}\;\kappa_{22}\right]}^{T}$, and

(10) $$I_{1}=\left[\begin{array}{cc} 1 & 0\\ 0 & 1\\ 0 & 0 \end{array}\right],I_{2}=\left[\begin{array}{cc} 0 & 0\\ 1 & 0\\ 0 & 1 \end{array}\right],e_{1}=\left\{\begin{array}{c} 1\\ 0 \end{array}\right\},e_{2}=\left\{\begin{array}{c} 0\\ 1 \end{array}\right\}.$$

### 2.3. Step 2: Strain Energy Expression

The strain energy of the sandwich panel can be expressed as

(11) $$U=\frac{1}{2}\int_{-b/2}^{b/2}\int_{-a/2}^{a/2}\frac{1}{\Omega }U_{\Omega }dx_{2}dx_{1},$$

where *a* and *b* are the length and width of the sandwich panel, respectively, $\frac{U_{\Omega }}{\Omega }$ is the stain energy density over the domain of the macroscopic plate, and can be expressed as

(12) $$U_{\Omega }=\int_{-\frac{h}{2}-t_{f}}^{-\frac{h}{2}}E^{b^{T}}C^{b}E^{b}dx_{3}+\int_{-\frac{h}{2}}^{\frac{h}{2}}E^{c^{T}}C^{c}E^{c}dx_{3}+\int_{\frac{h}{2}}^{\frac{h}{2}+t_{f}}E^{t^{T}}C^{t}E^{t}dx_{3}.$$

where the superscripts *b*, *c* and *t* denote the bottom facesheet, the core layer and the top facesheet, respectively, *h* and $t_{f}$ denote the core height and the thickness of the facesheet, respectively.

Equation (11) can be written as

(13) $$U=\frac{1}{2}\langle E^{T}DE\rangle =\frac{1}{2}\langle {\left\{\begin{array}{c} E_{e}\\ 2E_{s}\\ E_{t} \end{array}\right\}}^{T}\left[\begin{array}{ccc} D_{e} & D_{es} & D_{et}\\ D_{es}^{T} & D_{s} & D_{st}\\ D_{et}^{T} & D_{st}^{T} & D_{t} \end{array}\right]\left\{\begin{array}{c} E_{e}\\ 2E_{s}\\ E_{t} \end{array}\right\}\rangle ,$$

where $D_{e},D_{es},D_{et},D_{s},D_{st}$, and $D_{t}$ are the corresponding sub-matrices containing coefficients of the material stiffness matrix.

For the original sandwich panel, the virtual work carried out by the applied load is

(14) $$\begin{array}{c} W_{3D}=W_{2D}+W^{*}\\ \;=\int_{s}\left(p_{i}{\bar{u}}_{i}+q_{\alpha }\delta {\bar{u}}_{3,\alpha }\right)ds+\int_{s}\left(\langle f_{i}w_{i}\rangle +\tau_{i}h\varphi_{i}/2-\beta_{i}h\varphi_{i}/2\right)ds, \end{array}$$

where *s* denotes the reference surface; $-h\varphi_{i}/2$ and $h\varphi_{i}/2$ denote the warping displacements on the bottom and top surfaces of the sandwich panel, respectively; $f_{i}$ is the body force; $\beta_{i}$ and $\tau_{i}$ are the traction on the bottom and top surfaces, respectively; and the distributed forces and moments along the reference surface are defined as $p_{i}=\langle f_{i}\rangle +\tau_{i}+\beta_{i},q_{\alpha }=h/2\left(\beta_{\alpha }-\tau_{\alpha }\right)-\langle \zeta y_{3}f_{\alpha }\rangle$.

The kinetic energy of the panel is expressed as

(15) $$K=\frac{1}{2}\int_{\Omega }\rho v^{T}vd\Omega ,$$

where ρ is the equivalent density, *v* is the absolute velocity of a generic point in the panel, and can be expressed as

(16) $$v=V+\tilde{\omega }\left(\xi +w\right)+\dot{w},$$

where $\dot{w}=\partial w/\partial t$, *V* is the absolute velocity of a point in the deformed reference surface, $\tilde{\omega }$ is the inertial angular velocity, and $\xi ={\left[\begin{array}{ccc} 0 & 0 & x_{3} \end{array}\right]}^{T}$.

The original kinetic energy can be divided into kinetic energy of equivalent 2D plate and residual kinetic energy, such as

(17) $$K=K_{2D}+K^{*},$$

where

(18) $$K_{2D}=\frac{1}{2}\int_{s}\left(\bar{\mu }V^{T}V+2\omega^{T}\tilde{\mu \xi }V+\omega^{T}\Phi \omega \right)ds,$$

(19) $$K^{*}=\frac{1}{2}\int_{\Omega }\rho \left[{\left(\tilde{\omega }w+\dot{w}\right)}^{T}\left(\tilde{\omega }w+\dot{w}\right)+2{\left(V+\tilde{\omega }\xi \right)}^{T}\left(\tilde{\omega }w+\dot{w}\right)\right]d\Omega ,$$

with $\bar{\mu }=\langle \rho \rangle$, $\mu \xi ={\left\lfloor \begin{array}{ccc} 0 & 0 & \langle x_{3}\rho \rangle \end{array}\right]}^{T}$, and $\Phi =\left[\begin{array}{ccc} \langle x_{3}^{2}\rho \rangle & 0 & 0\\ 0 & \langle x_{3}^{2}\rho \rangle & 0\\ 0 & 0 & 0 \end{array}\right]$.

According to the Hamilton’s principle, the elastodynamic behavior of the plate is governed by

(20) $$\int_{t_{1}}^{t_{2}}\left[\delta \left(K_{2D}+K^{*}-U\right)+{\bar{\delta W}}_{2D}+{\bar{\delta W}}^{*}\right]dt=0,$$

where $t_{1}$ and $t_{2}$ are arbitrary fixed times.

The elastodynamic behavior of the panel is governed by Hamilton’s principle

(21) $$\int_{t_{1}}^{t_{2}}\left[\delta \left(K_{2D}+K^{*}-U\right)+{\bar{\delta W}}_{2D}+{\bar{\delta W}}^{*}\right]dt=0,$$

and $w_{i}$ can be solved from

(22) $$\min_{w_{i}\;\in \;\mathrm{Equation}\;\left(4\right)}\langle E^{T}DE\rangle .$$

Clearly, this variational problem is posed over the 3D unit cell only, and the solution of $w_{i}$ is the functions of the plate strains. The asymptotic analysis of the variational statement in Equation (21) can be used to solve the unknown warping function $w_{i}$ instead of making an ad hoc assumption, as discussed in the next section.

### 2.4. Step 3: Dimensional Reduction Analysis

To solve the unknown warping function $w_{i}$ using VAM, the order of each term in Equation (21) must be evaluated as

(23) $$\begin{array}{cc} \; & \epsilon_{\alpha \beta }∼h\kappa_{\alpha \beta }∼\varphi_{i}∼n,\;v_{i}∼hn,\;v_{∥;\alpha }∼w_{3;\alpha }∼\frac{h}{a}n\\ \; & v_{∥;3}∼v_{3;3}∼n,hf_{\alpha }∼\alpha_{\alpha }∼\beta_{\alpha }∼\mu \frac{h}{a}n,hf_{3}∼\alpha_{3}∼\beta_{3}∼\mu {\left(\frac{h}{a}\right)}^{2} \end{array},$$

where *n* is the order of the minimum strain and μ is the order of the material properties.

The zeroth-order approximation of the variational statement in Equation (21) can be obtained by removing the asymptotically smaller terms $K^{*}$ and ${\bar{\delta W}}^{*}$ according to VAM, such as

(24) $$\int_{t_{1}}^{t_{2}}\left[\delta \left(K_{2D}-\int_{\Omega }U_{0}d\Omega \right)+{\bar{\delta W}}_{2D}\right]dt=0,$$

where $U_{0}$ can be obtained dropping the derivatives with respect to $x_{\alpha }$ in Equation (13),

(25) $$2U_{0}=\langle \begin{array}{c} {\left(\epsilon +\zeta y_{3}\kappa \right)}^{T}D_{e}\left(\epsilon +\zeta y_{3}\kappa \right)+{\left(\varphi_{∥}+v_{∥,3}\right)}^{T}D_{s}\left(\varphi_{∥}+v_{∥,3}\right)\\ +2{\left(\epsilon +\zeta y_{3}\kappa \right)}^{T}\left(D_{es}\left(\varphi_{∥}+v_{∥,3}\right)+D_{et}\left(\varphi_{3}+v_{3,3}\right)\right)\\ +2{\left(\varphi_{∥}+v_{∥,3}\right)}^{T}D_{st}\left(\varphi_{3}+v_{3,3}\right)+{\left(\varphi_{3}+v_{3,3}\right)}^{T}D_{t}\left(\varphi_{3}+v_{3,3}\right) \end{array}\rangle .$$

The related Euler–Lagrange equation can be obtained by introducing Lagrange multipliers $\lambda_{i}$, such as

(26) $$\begin{array}{cc} \; & {\left[{\left(\epsilon +\zeta y_{3}\kappa \right)}^{T}D_{es}+{\left(\varphi_{∥}+v_{∥,3}\right)}^{T}D_{s}+\left(\varphi_{3}+v_{3,3}\right)D_{st}\right]}_{,3}=\lambda_{∥},\\ \; & {\left[{\left(\epsilon +\zeta y_{3}\kappa \right)}^{T}D_{et}+{\left(\varphi_{∥}+v_{∥,3}\right)}^{T}D_{st}+\left(\varphi_{3}+v_{3,3}\right)D_{t}\right]}_{,3}=\lambda_{3}, \end{array}$$

where $\lambda_{\left|\right|}={\left[\lambda_{1}\;\lambda_{2}\right]}^{T}$.

The free surface conditions are

(27) $$\begin{array}{cc} \; & {\left[{\left(\epsilon +\zeta y_{3}\kappa \right)}^{T}D_{es}+{\left(\varphi_{∥}+v_{∥,3}\right)}^{T}D_{s}+\left(\varphi_{3}+v_{3,3}\right)D_{st}^{T}\right]}^{+/-}=0\\ \; & {\left[{\left(\epsilon +\zeta y_{3}\kappa \right)}^{T}D_{et}+{\left(\varphi_{∥}+v_{∥,3}\right)}^{T}D_{st}+\left(\varphi_{3}+v_{3,3}\right)D_{t}\right]}^{+/-}=0 \end{array}$$

From these conditions, we can solve for $v_{\left|\right|}$ and $v_{3}$ as

(28) $$v_{∥}={\langle -\left(\epsilon +\zeta y_{3}\kappa \right){\bar{D}}_{es}{\left(D_{s}\right)}^{-1}\rangle }^{T},v_{3}=\langle -\left(\epsilon +\zeta y_{3}\kappa \right){\bar{D}}_{et}{\left({\bar{D}}_{t}\right)}^{-1}\rangle ,$$

where

(29) $$\begin{array}{c} {\bar{D}}_{es}=D_{es}-{\bar{D}}_{et}{\left(D_{st}\right)}^{T}{\left({\bar{D}}_{t}\right)}^{-1},\;{\bar{D}}_{et}=D_{et}-D_{es}{\left(D_{s}\right)}^{-1}D_{st},\\ {\bar{D}}_{t}=D_{t}-{\left(D_{st}\right)}^{T}{\left(D_{s}\right)}^{-1}D_{st}. \end{array}$$

Substituting Equation (27) in Equation (24), we obtain

(30) $$\begin{array}{c} U_{2D}=\frac{1}{2}\langle {\left(\epsilon +\zeta y_{3}\kappa \right)}^{T}K\left(\epsilon +\zeta y_{3}\kappa \right)\rangle =\frac{1}{2}{\left\{\begin{array}{c} \epsilon \\ \kappa \end{array}\right\}}^{T}\left[\begin{array}{cc} A & B\\ B^{T} & D \end{array}\right]\left\{\begin{array}{c} \epsilon \\ \kappa \end{array}\right\} \end{array},$$

where A,B, and D are 3×3 sub-matrices and can be expressed as

(31) $$\begin{array}{c} A=\langle K\rangle ,\;B=\langle \zeta y_{3}K\rangle ,\;D=\langle \zeta y_{3}^{2}K\rangle ,\\ K=D_{e}-{\bar{D}}_{es}D_{s}^{-1}D_{es}^{T}-{\bar{D}}_{et}D_{et}^{T}/{\bar{D}}_{t}. \end{array}$$

The constitutive relation of the equivalent 2D plate may be obtained as

(32) $$\left\{\begin{array}{c} N_{11}\\ N_{22}\\ N_{12}\\ M_{11}\\ M_{22}\\ M_{12} \end{array}\right\}=\left[\begin{array}{cccccc} A_{11} & A_{12} & A_{16} & B_{11} & B_{12} & B_{16}\\ A_{12} & A_{22} & A_{26} & B_{12} & B_{22} & B_{26}\\ A_{16} & A_{26} & A_{66} & B_{16} & B_{26} & B_{66}\\ B_{11} & B_{12} & B_{16} & D_{11} & D_{12} & D_{16}\\ B_{12} & B_{22} & B_{26} & D_{12} & D_{22} & D_{26}\\ B_{16} & B_{26} & B_{66} & D_{16} & D_{26} & D_{66} \end{array}\right]\left\{\begin{array}{c} \epsilon_{11}\\ \epsilon_{22}\\ 2\epsilon_{12}\\ \kappa_{11}\\ \kappa_{22}\\ 2\kappa_{12} \end{array}\right\}.$$

where $N_{\alpha \beta }=\frac{\partial U_{2D}}{\partial \epsilon }$, $M_{\alpha \beta }=\frac{\partial U_{2D}}{\partial \kappa }$.

The variational statement in Equation (24) only involves the 2D field variables with macro-coordinates $x_{\alpha }$. As a result, the obtained equivalent 2D plate (2D-EPM) can be used to replace the original sandwich panel in the global analysis, such as global displacement and buckling, and can be solved using a linear solver in finite element software like ABAQUS/Standard.

### 2.5. Step 4: Recovering Relations from 2D to 3D

The accuracy in recovering the local fields of the original three-dimensional SP-PSC determines the reliability of the equivalent 2D plate model. As a result, the recovery relationship must be provided. In other words, the 2-D variables and warping functions are used to describe the 3D local fields. The local displacement field can be recovered according to Equation (4), such as

(33) $$u_{i}={\bar{u}}_{i}+\left[\begin{array}{ccc} {\bar{u}}_{1,1} & {\bar{u}}_{1,2} & {\bar{u}}_{1,3}\\ {\bar{u}}_{2,1} & {\bar{u}}_{2,2} & {\bar{u}}_{2,3}\\ {\bar{u}}_{3,1} & {\bar{u}}_{3,2} & {\bar{u}}_{3,3} \end{array}\right]\left\{\begin{array}{c} y_{1}\\ y_{2}\\ y_{3} \end{array}\right\}+\zeta \left(y_{3}\varphi_{i}+v_{i}\right),$$

The local strain field can be recovered as

(34) $$E_{e}^{0}=\epsilon +x_{3}\kappa ,\;2E_{s}^{0}=-\varphi_{\left|\right|},\;E_{t}^{0}=\varphi_{3}+v_{3,3}.$$

According to the constitutive relations, the local stress field can be recovered as

(35) $$\sigma_{e}=D_{e}E_{e}+D_{et}E_{t},\;\sigma_{s}=D_{s}\left(2E_{s}\right),\;\sigma_{t}=D_{et}^{T}E_{e}+D_{t}E_{t}$$

The equivalent density of the SP-PSC can be calculated as

(36) $$\rho =\frac{m}{V_{\Omega }}=\frac{8\times S_{c}h\rho_{c}+4\times \left(l_{1}+l_{2}\right)th\rho_{c}+2\times l_{3}th\rho_{c}+2abt_{f}\rho_{f}}{ab\left(h+2t_{f}\right)}$$

where $\rho_{f}$ and $\rho_{c}$ are the densities of the face sheet and core layer, respectively; $S_{c}$ is the arc length of the petal wall.

## 3. Model Validation

**Figure 2:**
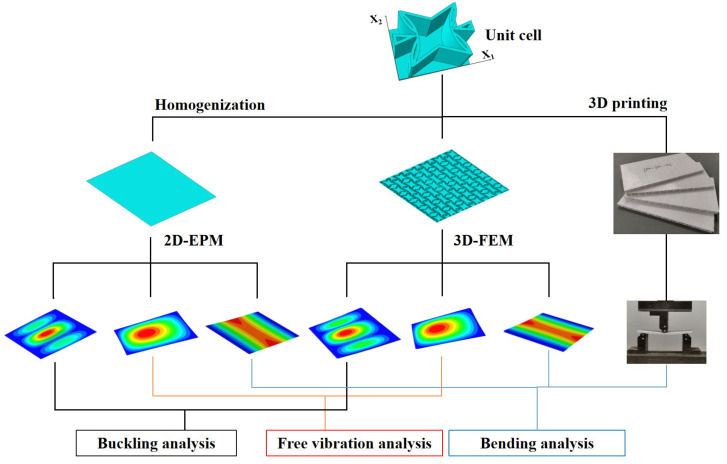
Verification flowchart of the accuracy of 2D-EPM for SP-PSC.

The accuracy of 2D-EPM is validated in this section by comparing it to experimental data, as well as bending, buckling, and free vibration results from 3D-FEM under various boundary and load conditions, as shown in Figure 2. The 3D-FEM of SP-PSC is created by repeating the unit cell with dimensions of 20mm×15mm along the $x_{1}$ and $x_{2}$ directions. The size of the macro plate is 240mm×135mm in the three-point bending test, while 200 mm×200mm in other cases. Since the VAM-based 2D-EPM is developed by taking advantage of small parameters, the global size of the plate should be close to or greater than 10 times the cell size, resulting in more accurate numerical simulation results. The SP-PSC is composed of top and bottom facesheets with a thickness of 0.5 mm and a core layer with a height of 9 mm. The material properties are from the 3D printing materials: $E=2100\;\mathrm{MPa},\;\rho =1300\;\mathrm{kg}\cdot m^{-3}$ and μ=0.41. In the ABAQUS package, the material parameters are input in the “property” option of “module” and assigned to the established model. The boundary and loading conditions of the model are defined in the “load” option of “module”. The relative error between 2D-EPM and 3D-FEM is defined as: Error = ∣2D-EPM results −3D-FEM results ∣/3D-FEM results (%).

**Figure 3:**
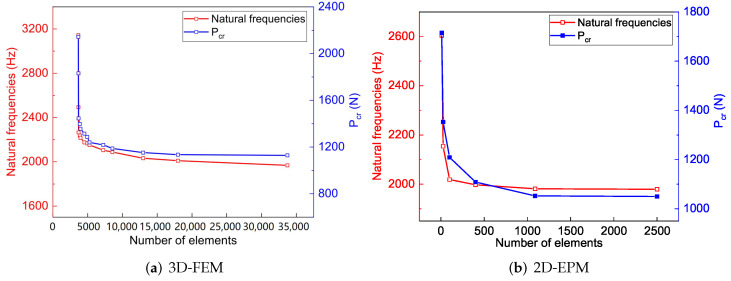
Mesh convergence study for 3D-FEM and 2D-EPM.

The mesh convergence has been studied before the numerical simulation, and the results are shown in Figure 3. For 3D-FEM, the numerical simulation had excellent convergence when the number of elements was more than 15,000. Although adding more elements can improve the accuracy of the numerical simulation, it also puts forward higher requirements for the calculated cost. Therefore, 324,869 C3D20R elements for 3D-FEM were adopted to maximize computation efficiency. Similarly, a mesh convergence study for 2D-EPM was also conducted. The study found that the numerical simulation had good convergence when the number of elements was more than 1000, and 10,201 S4R elements for 2D-EPM were adopted based on computation efficiency. In addition, the mesh of the contact surface between the loading head and the surface was refined during the 3-point loading simulation.

### 3.1. Static Deflection Analysis

The SP-PSC has the characteristics of light weight and high strength. Therefore, it is necessary to study the bending performance of SP-PSC accurately and quickly. In this sub-section, the WDW-10 electronic universal testing machine with a maximum load of 10 kN was used to perform the three-point bending test. A constant loading with a speed of 5 mm/min was adopted in the test, and the displacement of the central roll was recorded. Considering that the heat dissipation of the 3D printing process was slow due to the high temperature, a non-closed 3D printer with a single nozzle was used to print the samples.

**Figure 4:**
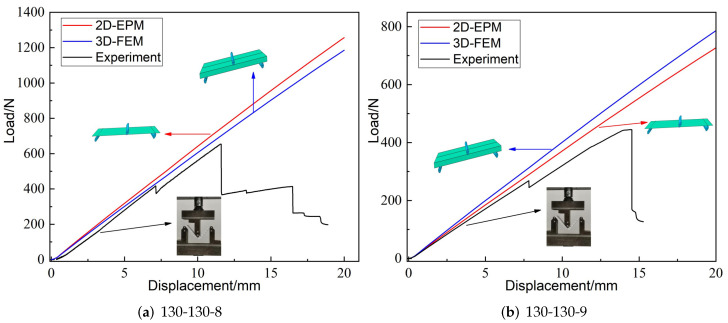

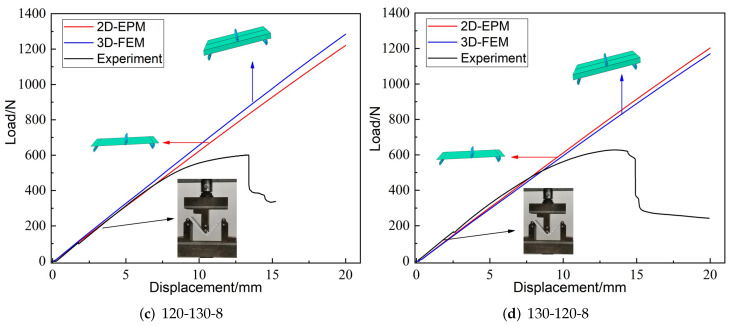
Displacement-load curves from experiments, 2D-EPM, and 3D-FEM, unit: mm ($\theta_{1}$-$\theta_{2}$-*h* denote the included angle between cancave petal walls, included angle between cancave inclined walls and core height, respectively).

Figure 4 shows the displacement-load curves from experiments, 3D-FEM and 2D-EPM under three-point bending load. It can be seen that the linear elastic lines predicated by 3D-FEM and 2D-EPM were very similar to the curves obtained from experiments. In addition, the slope errors corresponding to the three linear elastic stages were less than 10%, meeting the requirements of engineering accuracy. The sources of the errors mainly included two aspects: (a) the bottom facesheet and the core layer were printed together, while the top facesheet was printed separately, resulting in a decrease in the overall mechanical performance; (b) the temperature affected the forming quality of the printed sandwich panel and the bearing performance under loading.

**Figure 5:**
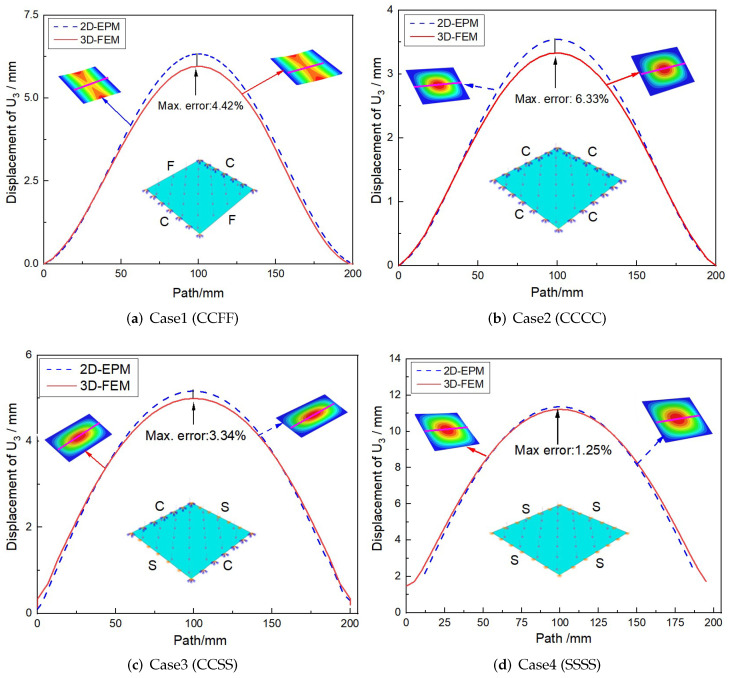
Comparison of deflection curves along the center line obtained by 2D-EPM and 3D-FEM under different BCs (the indices C, S, and F represent clamped, simply supported, and free boundary conditions, respectively).

Figure 5 shows the deflection curves along the center line obtained by 3D-FEM and 2D-EPM under four BCs and a uniformly distributed load of $10\;N/{\mathrm{mm}}^{2}$. It can be seen that the deflection corresponding to the center line was larger as it was farther away from the boundary. At the same time, the displacement obtained by 3D-FEM under four BCs was less than that obtained by 2D-EPM, which may be due to the different meshing between 2D-EPM and 3D-FEM. The deflection error under different BCs was less than 7%, which also meets the requirements of engineering accuracy.

### 3.2. Global Buckling Analysis

The sandwich panel is prone to buckling when subjected to an in-plane load. This section mainly compares the global buckling modes and buckling loads of 2D-EPM and 3D-FEM under the same boundary conditions. The loads applied to the boundary of 2D-EPM and 3D-FEM are 1N/mm and 1×200=200N, respectively.

Table 1 shows the first four buckling modes of SP-PSC under three different boundary conditions, and the numbers in parentheses indicate the relative error of the buckling loads. It can be found that the error of the buckling loads was less than 6%. At the same time, it can be found that the buckling modes of 2D-EPM and 3D-FEM were almost the same under different boundary conditions, indicating that 2D-EPM can replace 3D-FEM in the global buckling analysis of SP-PSC with confidence.

### 3.3. Free Vibration Analysis

Table 2 shows the first four natural frequencies predicted by 2D-EPM and 3D-FEM under different BCs. Their natural frequencies were in good agreement. The errors of natural frequencies increased with an increase in orders, but they were all within 7%. It is worth noticing that the natural frequency increased gradually with the enhancement of boundary constraints. For example, the fundamental frequency under the CCCC BCs (669.6 Hz) was 1.5 times that under the SSSS BCs (487.3 Hz).

Table 3 shows the first eight free vibrations of SP-PSC under the CCCC boundary condition. It can be found that the error of natural frequency was within 7% regardless of order, low or high. At the same time, the vibration modes predicted by the two models were identical in all orders. There was only one half-wave along the $x_{2}$ direction in modes 1 and 3; two half-waves along the $x_{2}$ direction in modes 2, 4 and 8; and three half-waves along the $x_{2}$ direction in mode 7. On the contrary, there was only one half-wave along the $x_{1}$ direction in modes 1 and 2, two half-waves along the $x_{1}$ direction in modes 3, 4 and 7, and three half-waves along the $x_{1}$ direction in mode 8. The mode shapes of modes 5 and 6 were axisymmetric or centrosymmetric, which was related to the consistency of the density gradient of the sandwich panel along the direction of $x_{1}$ and $x_{2}$.

### 3.4. Comparison of Calculation Efficiency

Table 4 shows that the 2D-EPM has three advantages over the 3D-FEM: (1) the definition of contact, the application of the load, and the boundary constraints are more convenient and concise; (2) the different meshing of the 3D-FEM has a greater impact on the calculation speed and accuracy; whereas the meshing of the 2D-EPM is faster and less difficult; and (3) the calculation efficiency of 2D-EPM is nearly 20 times higher than that of 3D-FEM. The configuration includes a Lenovo XiaoXinAir 15 ITL powered by an 11th Gen Intel i5-1135G7 CPU running at 3.2 GHz and 32 GB of RAM.

## 4. Variable Parameter Analysis

**Figure 6:**
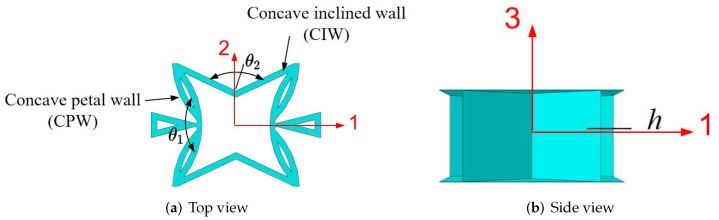
Geometric parameters of unit cell within the SP-PSC.

The SP-PSC is an anisotropic structure, which means that it has different effective performances in different directions. As a result, it is critical to investigate the change in effective performance with different geometric and material parameters. The geometric parameters of SP-PSC shown in Figure 6 mainly include: the included angle $\theta_{1}$ between concave petal walls (CPW for short), the included angle $\theta_{2}$ between concave inclined walls (CIW for short), wall thickness of core layer (*t*), core height (*h*) and plate height (*H*). The geometric and material parameters in Section 3 were used as benchmark parameters except that the facesheet thickness was set to 0.075 mm to demonstrate the NPR effect. The geometric parameters of the benchmark model are $130^{\circ }-130^{\circ }-9.85$ ($\theta_{1}-\theta_{2}-h$).

### 4.1. Included Angles $\theta_{1}$

**Figure 7:**
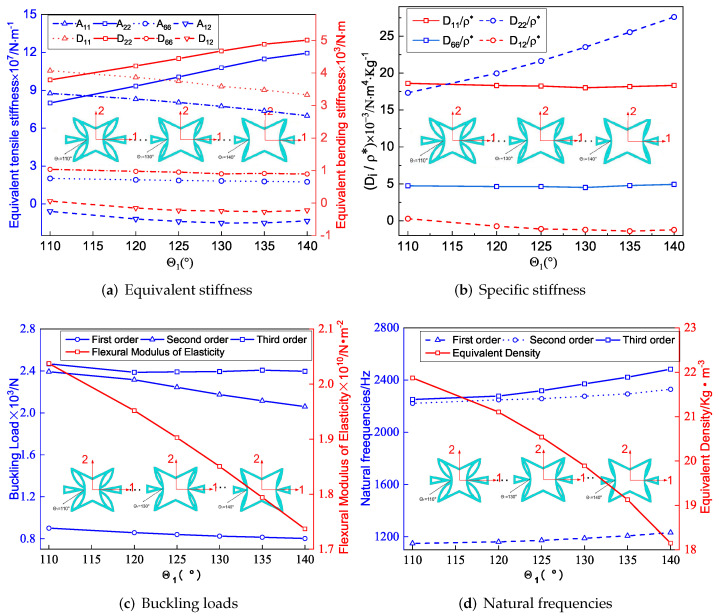
Influence of included angle $\theta_{1}$ on equivalent stiffness of SP-PSC.

Figure 7a shows the effect of included angle $\theta_{1}$ on the equivalent stiffness of SP-PSC. It can be observed that the equivalent tensile stiffness $A_{22}$ and equivalent bending stiffness $D_{22}$ increased as $\theta_{1}$ increased, while other stiffness decreased. The deformation of the core cell along the $x_{2}$ direction was mainly caused by the axial deformation of the CPW when $\theta_{1}$ approached $180^{\circ }$, but there was no external constraint at the intersection of the CPW when $\theta_{1}<180^{\circ }$. Therefore, the deformation of the core layer along the $x_{2}$ direction was mainly caused by the axial and rotation deformations of CPW (which was also the reason for the NPR effect), and this rotation tended to increase the deformation rather than weaken the deformation. Therefore, the larger the $\theta_{1}$, the smaller the deformation of the core along the $x_{2}$ direction, resulting in an increase in $A_{22}$. In addition, there was a synchronous change between the equivalent bending and tensile stiffness in the corresponding direction.

Figure 7b shows the variation of specific stiffness when $\theta_{1}$ changed. It can be found that $D_{11}/\rho^{*}$ increased significantly with the increase of $\theta_{1}$. As a result, the smaller $\theta_{1}$, the greater the advantage of SP-PSC. It is worth noting that the included angle $\theta_{1}$ had little effect on the other three specific stiffnesses. Figure 7c,d show the influence of $\theta_{1}$ on the buckling load and natural frequency of SP-PSC. The equivalent flexural elastic modulus of the sandwich panel decreased with the increase in $\theta_{1}$, so the anti-buckling capacity also decreased. It can be found that the equivalent density of the plate decreased in a small range with an increase in $\theta_{1}$, resulting in an increase in natural frequency, which may be related to the small increase in equivalent stiffness.

### 4.2. Included Angles $\theta_{2}$

**Figure 8:**
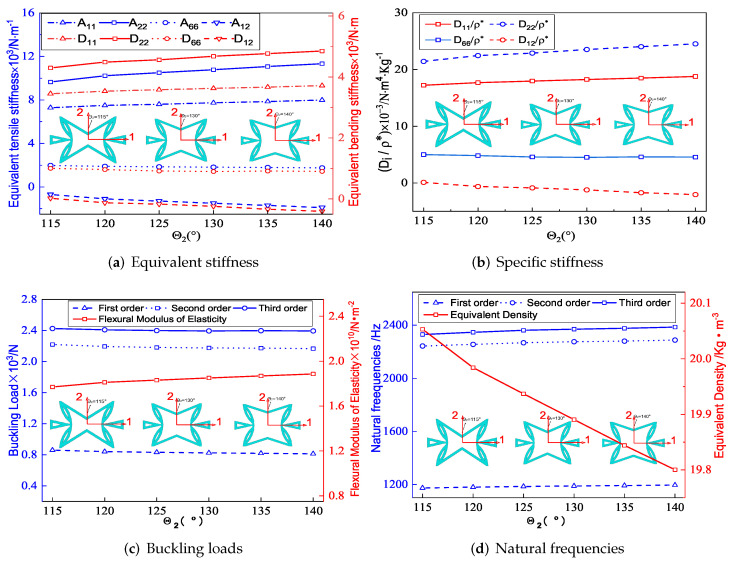
Influence of included angle $\theta_{2}$ on effective performance of SP-PSC.

Figure 8a shows that the equivalent tensile stiffness $A_{11}$, $A_{22}$ and the equivalent bending stiffness $D_{11}$, $D_{22}$ increased with the increase of $\theta_{2}$, but the increase in $A_{22}$ and $D_{22}$ was relatively obvious. The CPW of the core cell remained unchanged when $\theta_{2}$ changed. The CIW was parallel to the $x_{1}$ direction when $\theta_{2}=180^{\circ }$. At that time, the deformation along $x_{1}$ direction was primarily caused by the axial deformation of CIWs when loaded in the same direction. However, as $\theta_{2}$ was gradually decreased, the concave degree of CIWs increased, and the deformation in the $x_{1}$ direction was gradually dominated by the additional deformation caused by rotation. As a result, as $\theta_{2}$ decreased, the deformation in the $x_{1}$ direction increased, indicating that the equivalent stiffness of $A_{11}$ and $D_{11}$ of SP-PSC decreased as $\theta_{2}$ decreased.

**Figure 9:**
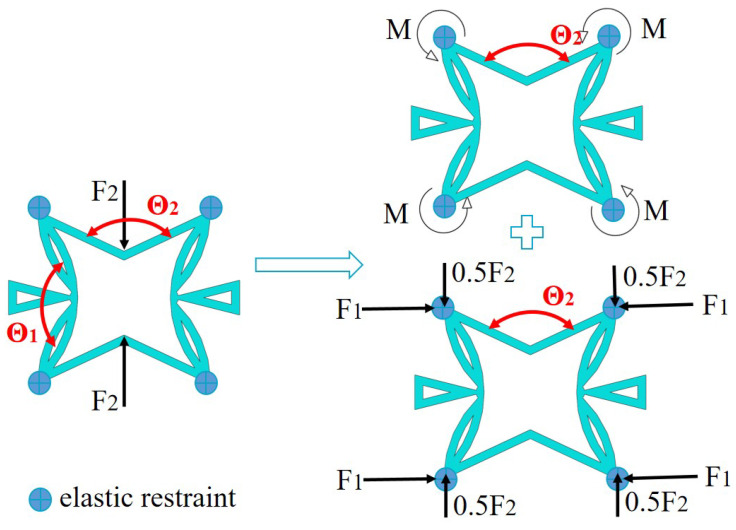
Simplified model for analyzing the deformation mechanism of core cell withint the SP-PSC.

To study the deformation mechanism of the core layer in SP-PSC, a core cell loaded only in the $x_{2}$ direction was considered as shown in Figure 9, in which the four corners of the core cell were elastically restrained. There were three different actions when the load was transferred to the corner through CIWs: the bending moment in the left and right CPWs; $0.5\;F_{2}$ in the $x_{2}$ direction; and $F_{1}$ in the $x_{1}$ direction.

The left and right CPWs under $0.5\;F_{2}$ load generated compression deformation along the $x_{2}$ direction while generating tensile deformation along the $x_{2}$ direction under $F_{1}$ load. As a result, the action of $F_{1}$ reduced the deformation caused by $0.5\;F_{2}$ in the $x_{2}$ direction. The included angle between CIW and $x_{2}$ direction increased as $\theta_{2}$ increased. That is, when the core cell was subjected to the action of $F_{2}$, the value of $F_{1}$ became larger, so the deformation in the corresponding $x_{2}$ direction was smaller. It can be concluded that the equivalent stiffness of $A_{22}$ and $D_{22}$ increased with the increase in $\theta_{2}$.

Figure 8b shows the effect of $\theta_{2}$ on specific stiffness. As $\theta_{2}$ increased, $D_{11}/\rho^{*}$ and $D_{22}/\rho^{*}$ increased, while $D_{12}/\rho^{*}$ decreased. Hence, the SP-PSC with the larger the $\theta_{2}$ should be selected to make full use of the performance of the panel. Figure 8c,d show the effects of included angle $\theta_{2}$ of CIWs on buckling load and natural frequency of SP-PSC. It can be found that the influence of $\theta_{2}$ on the anti-buckling capacity was not very significant, while the natural frequency of SP-PSC increased. The reason may be due to the fact that the equivalent density of SP-PSC gradually decreased, and the stiffness $D_{11}$ and $D_{22}$ gradually increased with the increase of $\theta_{2}$.

### 4.3. Height Ratio h/H

**Figure 10:**
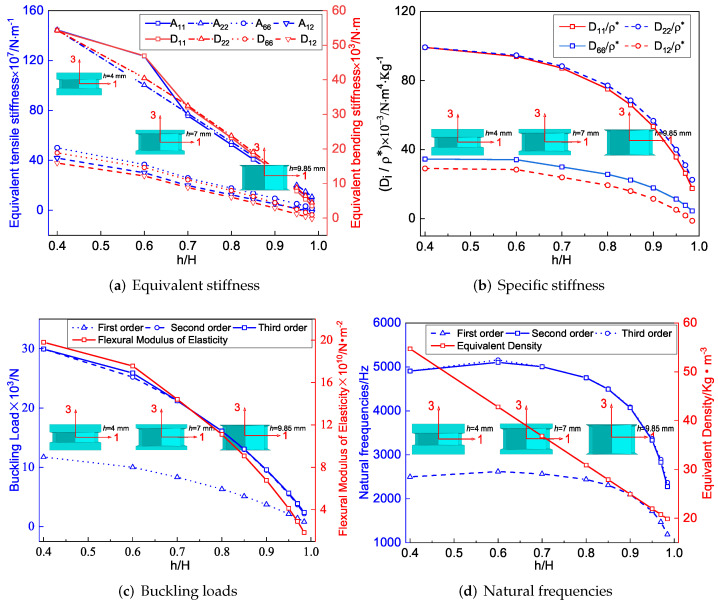
Influence of included angle $\theta_{2}$ on effective performance of SP-PSC.

Figure 10a shows the effect of the height ratio h/H on equivalent stiffness when all other geometric and material parameters are held constant. It can be found that when the total height of SP-PSC remained unchanged, the equivalent tensile stiffness gradually decreased with the increase in h/H. This change was straightforward to comprehend since the tensile stiffness of the core layer was weaker than that of the facesheet with the same thickness. It is worth noting that the change of equivalent bending stiffness was consistent with that of equivalent tensile stiffness.

Figure 10b shows the specific stiffness of SP-PSC decreased as h/H increased, but the change in h/H had little effect on the specific stiffness when h/H<0.6. The reduction of specific stiffness increased when h/H>0.6. Therefore, from an economic point of view, SP-PSC with h/H<0.6 should be used in actual applications to the greatest extent feasible. Figure 10c,d show the influence of height ratio on the buckling load and natural frequency of SP-PSC. Because the equivalent flexural elastic modulus reduced as the height ratio increased, so did the anti-buckling capacity, particularly the high-order anti-buckling ability. The equivalent density of the plate reduced linearly as the height ratio increased, and so did the equivalent stiffness. The reduction of the equivalent stiffness had a greater impact on the natural frequency than the equivalent density, so the natural frequency first increased slightly and then decreased greatly when h/H>0.6, which should be paid attention to in structural design.

## 5. Negative Poisson’s Ratio

This section studies the variation of the NPR of SP-PSC by changing geometric and material parameters, which lays a foundation for improving the NPR effect of SP-PSC for practical application.

### 5.1. SP-PSC with Isotropic Materials

The facesheet and core layer of SP-PSC in this section were made of isotropic materials, namely carbon steel (E=210 GPa, μ=0.3). The effect of geometric parameters of the core layer $(\theta_{1},\theta_{2}$ and h/H) on the NPR of SP-PSC was investigated.

**Figure 11:**
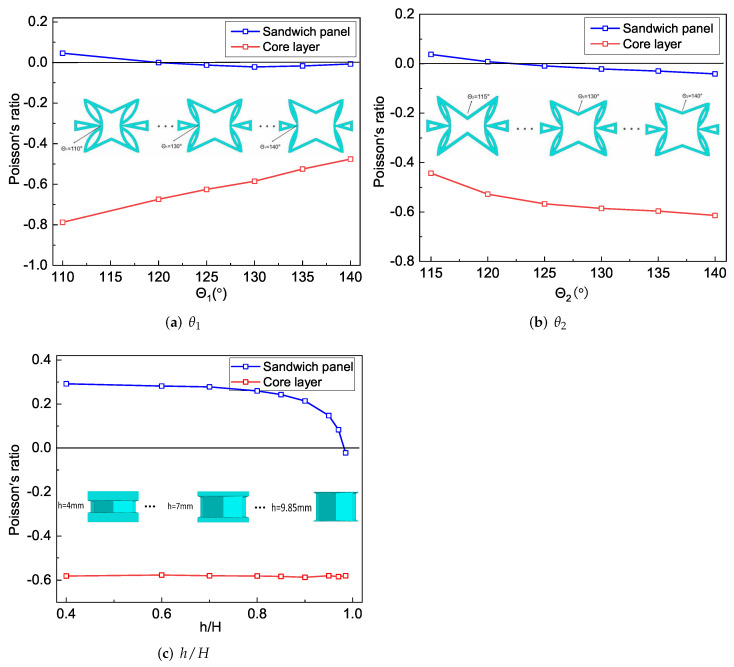
Influence of geometric parameters on the NPR of SP-PSC.

Figure 11a shows the changes in Poisson’s ratio of the core layer and the sandwich panel when $\theta_{1}$ changed. It can be found that the NPR effect of the core layer gradually weakened with the increase in $\theta_{1}$. The CPWs had a significant NPR effect relative to the non-concave walls. The CPWs tended to be parallel to the $x_{2}$ direction when $\theta_{1}$ gradually increased, resulting in the absolute value of the NPR of the core gradually decreasing. The sandwich panel has no NPR effect when $\theta_{1}<120^{\circ }$, but it has an increasing NPR effect when $120^{\circ }<\theta_{1}<130^{\circ }$ and a decreasing NPR effect when $130^{\circ }<\theta_{1}<140^{\circ }$.

It can be found from Figure 7a that the tensile stiffness $A_{22}$ of SP-PSC increased when $120^{\circ }<\theta_{1}<140^{\circ }$. The load carried by the core layer along the $x_{2}$ direction gradually increased with the increase in $\theta_{1}$, indicating that the influence of the core layer deformation was greater than that of the facesheet deformation. However, the NPR effect of the core layer gradually weakened with the increase in $\theta_{1}$. Under the combined action of these two factors, the Poisson’s ratio of the sandwich panel had a critical point at $\theta_{1}=130^{\circ }$, as shown in Figure 11a.

Figure 11b shows that the NPR of the core layer and sandwich panel gradually increased as $\theta_{2}$ increased. The NPR effect of the core layer gradually increased with the increase of $\theta_{2}$, and the increase in $\theta_{2}$ led to the gradual reduction in the stiffness of CIWs in the $x_{2}$ direction. Therefore, when the core layer was loaded in the $x_{2}$ direction, the larger the $\theta_{2}$, the higher the load proportion undertaken by CPWs, improving the NPR effect of the core layer.

The NPR effect of the sandwich panel gradually increased with an increase in $\theta_{2}$, which was mainly due to two aspects: (1) the equivalent stiffness $A_{22}$ of SP-PSC increased as $\theta_{2}$ increased in Figure 8a, when the SP-PSC was loaded in the $x_{2}$ direction, the weight of the load undertaken by the core layer increased, and the influence of the deformation of the core layer on the deformation of the SP-PSC was higher than that of the panel; (2) the absolute value of the NPR of the core layer gradually increased as $\theta_{2}$ increased. The Poisson’s ratio of SP-PSC was more sensitive to changes in $\theta_{2}$ than $\theta_{1}$.

Figure 11c shows the effects of different height ratios on the NPR of the core layer and the sandwich panel. It can be found that the change of h/H had little effect on the NPR of the core layer. However, with an increase in h/H, the Poisson’s ratio of SP-PSC gradually changed from a positive Poisson’s ratio to an NPR. The reason could be that the tensile stiffness $A_{22}$ of the facesheet gradually decreased as *h* decreased, whereas that of the core layer gradually increased, resulting in a gradual increase in the effect weights of core layer deformation in the overall deformation of SP-PSC.

### 5.2. SP-PSC with Composite Laminated Facesheets

In this section, the top/bottom facesheet of SP-PSC is made of heterogeneous material CFPR, and material parameters were: $\rho =1.49\;g/{\mathrm{cm}}^{3}$, $E_{1}=105.5$ GPa, $E_{2}=E_{3}=11.3$ GPa, $G_{12}=$ $G_{13}=3.23$ GPa, $G_{23}=3.18$ GPa, $\mu_{12}=\mu_{13}=0.28$, and $\mu_{23}=0.53$. The constraints of CFRP facesheets on the core layer changed throughout the process of collaborative deformation of the facesheets and the core layer. As a result, the composite laminated facesheet was expected to affect the NPR of SP-PSC.

**Figure 12:**
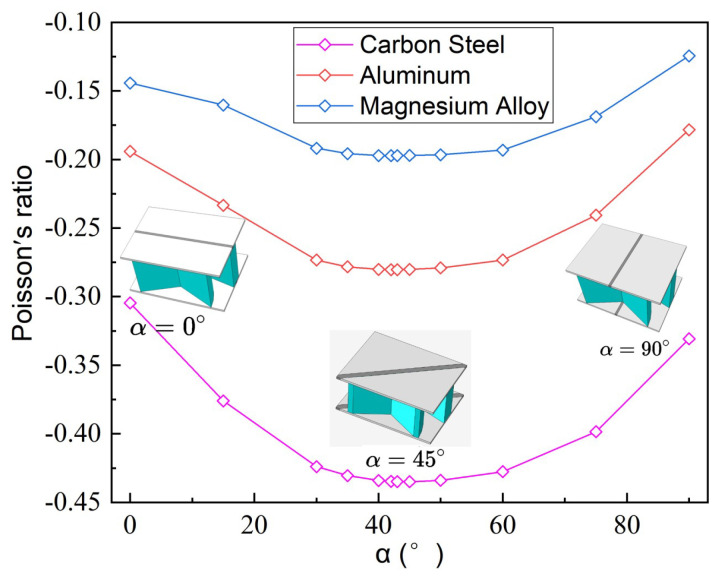
Variations of the negative Poisson’s ratio of SP-PSC with different core materials and unidirectional fiber angles.

To more conveniently study the influence of composite anisotropy on the NPR of SP-PSC, the top/bottom facesheets were made of 4-layer CFRP unidirectional laminates with a thickness of 0.075mm, and the core layer was made of isotropic materials: carbon steel (E=210 GPa, μ=0.3), aluminum (E=70 GPa, μ=0.3) and Magnesium Alloy (E=45 GPa, μ=0.35). Figure 12 shows the variations of NPR with different fiber angles (defined as the angle between the fiber direction and $x_{1}$ axis) of the top and bottom laminated facesheet. The NPR increased first and then decreased gradually with increasing fiber angle α, and these changes remained when the material properties of the core layer changed, indicating that the effect of fiber angle on the NPR had little relationship with the material used in the core layer. In addition, the NPR effect was shown to be most significant when the fiber angle of unidirectional composite laminates was in the range of 40∼$50^{\circ }$.

The direction of reinforcement fiber was parallel to the CIW of the unit cell when the fiber angle α equals $90^{\circ }$. That is, the carbon fiber reinforcement limited the relative displacement of the CIWs. Because the load along the $x_{2}$ direction was mostly transmitted through CIW in the unit cell, the load undertaken by CPW was reduced, resulting in a reduction in the NPR effect. The direction of reinforcement fiber was parallel to the CPW of the unit cell when $\alpha =0^{\circ }$. The load undertaken by CPW increased, but the laminated facesheet also directly limited the relative displacement of the CPWs. The NPR effect was weakened as a result of the limited relative displacement between CPWs.

**Figure 13:**
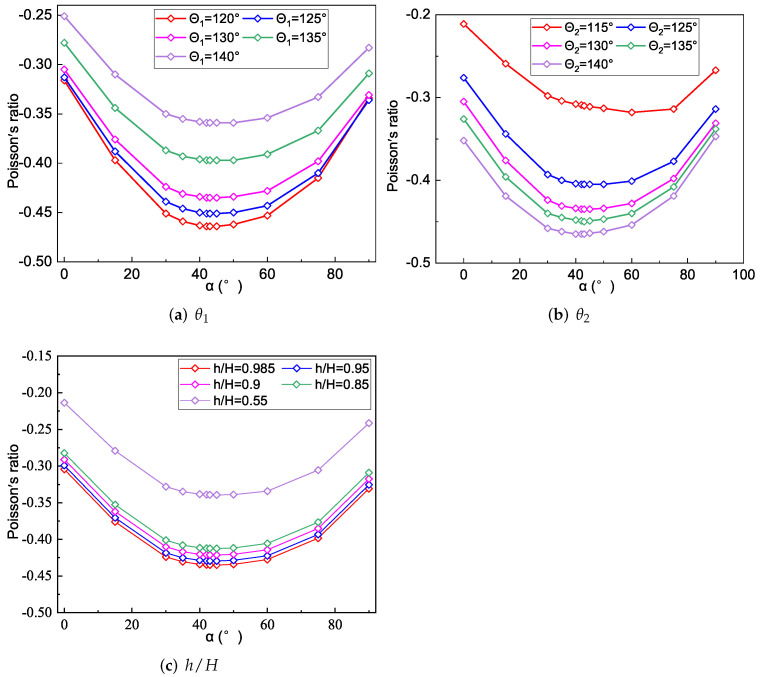
Effects of $\theta_{1},\theta_{2}$ or h/H on the NPR of SP-PSC.

To study the relationship between the NPR and fiber angle α, we investigated the variations of α-NPR curves by changing the geometric parameters $\theta_{1},\theta_{2}$ and *h* of core layer, respectively. Figure 13 shows that the NPR of SP-PSC with different $\theta_{1},\theta_{2}$ or *h* increased first and then decreased as α increased. The NPR of the sandwich panel was most significant when the fiber angle α was between $40^{\circ }$ and $50^{\circ }$, which was similar to the results in Section 5.2. In addition, when the top and bottom facesheets of SP-PSC were made of fiber reinforced composites, the NPR decreased with an increase in $\theta_{1}$. The NPR effect increased with an increase in $\theta_{2}$, and the NPR of the sandwich panel increased with the core height *h*.

## 6. Comparative Analysis

### 6.1. PSC, STC and SC

**Figure 14:**
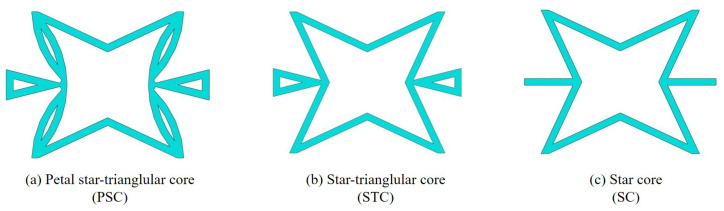
Core cell of petal star-triangular core, star-triangular core and star core.

The PSC evolved from the star-triangular core (STC) as shown in Figure 14. These two honeycombs are actually variations of the star core (SC) proposed by Theocaris et al. [9] and the improved star honeycomb proposed by Wei et al. [30]. This section compares the sandwich panels with three honeycombs in four aspects: equivalent stiffness, buckling, free vibration, and NPR effect. The 2D-EPM with a dimension of 200 mm × 200 mm was used to simulate the buckling and free vibration behaviors under SSSS and CCCC boundary conditions, respectively.

Table 5 shows the recovered local stress, strain, and displacement fields at the midpoint of 2D-EPM for PSC, STC, and SC in case 2, respectively. The top facesheet was removed for a better view. The local stress, strain, and displacement of PSC were smaller than those of STC and SC, and the local stress and strain of PSC were evenly distributed between the core layer and the facesheet due to the fact that the petal walls of PSC increased the bonding area with the facesheet. The connection between the core layer and facesheets was also poor in STC, but the local deformation was small, and the uniformity of local stress and strain was better than in SC due to the sufficient structural stiffness.

**Figure 15:**
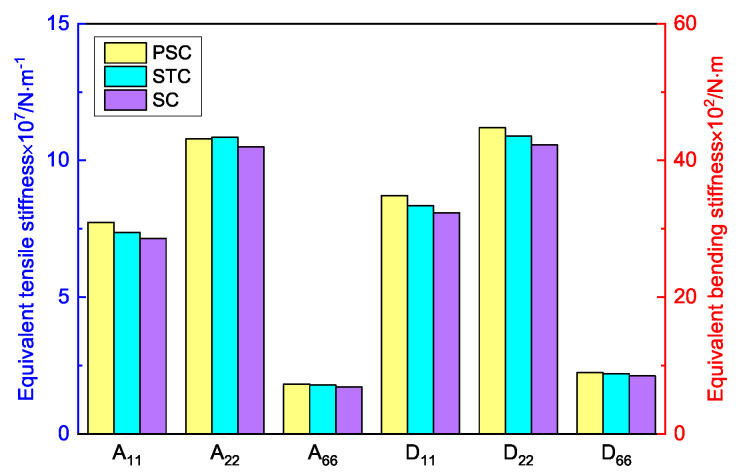
Comparison of equivalent stiffness between three sandwich panels with different honeycomb cores (PSC, STC and SC).

Figure 15 shows the equivalent stiffness of the three panels in different directions. There was little difference in the equivalent stiffness of the three panels, but the equivalent stiffness of SP-PSC was higher. It can be seen that replacing the horizontal link with a triangular link can improve the equivalent stiffness, while changing the concave inclined walls into arc walls can further improve the equivalent stiffness of the panel, which is of great significance in engineering.

**Figure 16:**
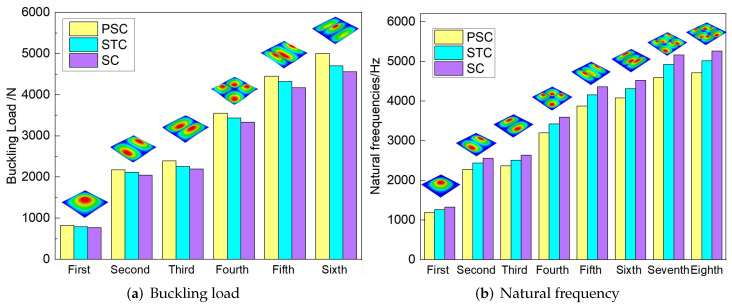
Comparison of the buckling load and natural frequency of three sandwich panels with different honeycomb cores.

**Figure 17:**
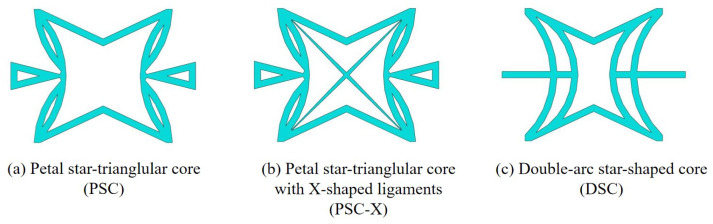
Core cell of (**a**) petal star-triangular core, (**b**) petal star-triangular core with X-shaped ligaments and (**c**) double-arc star-shaped core.

Figure 16 compares the first six buckling loads and the first eight natural frequencies of three sandwich panels with different honeycomb cores. The first six buckling loads of SP-PSC were greater than those of the other two sandwich panels, indicating that the anti-buckling capacity of the proposed SP-PSC was better than the other two panels. The changes in the first eight natural frequencies shown in Figure 17b were exactly the opposite of the anti-buckling ability. It can be seen from the structural analysis that whether replacing the horizontal link with a triangular link or replacing the concave linear walls with arc walls, the equivalent stiffness of the panel increased and the specific stiffness was reduced. Therefore, the specific stiffness of PSC should be improved as much as possible in the following section.

Table 6 compares the Poisson’s ratio of the core layer and the whole panel with different honeycomb cores. It can be found that the Poisson’s ratio of the three core layers was negative. The relationship between the Poisson’s ratio of the core layer and the whole panel was consistent. The NPR of SP-PSC was the largest, followed by SP-STC, and that of SP-SC was the smallest. Therefore, the hardness and shear capacity of SP-PSC were the strongest, followed by SP-STC and SP-SC. It can be seen that the stiffness, hardness, shear capacity, and NPR effect of SP-PSC were optimal except for specific stiffness.

### 6.2. PSC, PSC-X and DSC

The proposed SP-PSC has excellent NPR, but there is potential for improvement in some aspects. The core cell of SP-PSC, which is similar to novel re-entrant circular auxetic honeycombs [30], is easy to crack at the intersection of the left and right concave annular members before the overall bearing capacity of the panel has been fully developed. In addition, the stress in this small area is complex and relatively concentrated, so the initial defects of the panel have an extremely adverse impact. To address this issue, the DSC model is proposed on the basis of PSC by increasing the intersection area and reducing the complexity of the intersection stress as shown in Figure 17. The horizontal outriggers on both sides of the PSC are triangular, mainly considering that the constraints on both ends are not strong. To improve the critical bearing capacity, it is specially set as a variable cross-section in the design. However, the horizontal outriggers on both sides of the improved DSC are changed into thinner constant cross-sections because the constraints on both ends of the DSC outrigger are significantly enhanced.

To address the above issues, another improved scheme is proposed, PSC-X. In the field of civil engineering, the stiffness of the structure is often increased by adding X-shaped support. Inspired by this, the X-shaped support was added on the basis of PSC. The support shares the load undertaken by the left and right CIMs, and improves the overall stiffness of SP-PSC. Compared with PSC, the local stresses of the two improved cores at the intersection of the left and right CPMs have been reduced, which is what we expected. In addition, the specific stiffness of improved cores increases compared with PSC.

Based on the principle of control variables, the cell sizes of both improved models are kept consistent with PSC. That is, the top and bottom facesheets are both 0.075 mm thick, the wall thickness of the core layer is 0.7 mm, the lengths of the unit cells in $x_{1}$ and $x_{2}$ directions are 20 mm and 15 mm, respectively, and the height of the panel is 10 mm. The elastic modulus *E* of isotropic steel is 210,000 MPa, the Poisson’s ratio μ=0.3, and the density $\rho =7850\;\mathrm{kg}/m^{3}$. The dimensions of the panels in the comparison process are 200mm×200mm×10mm, of which the thicknesses of the top and bottom facesheets of the three panels are 0.075 mm and the core layer is 9.85 mm.

**Figure 18:**
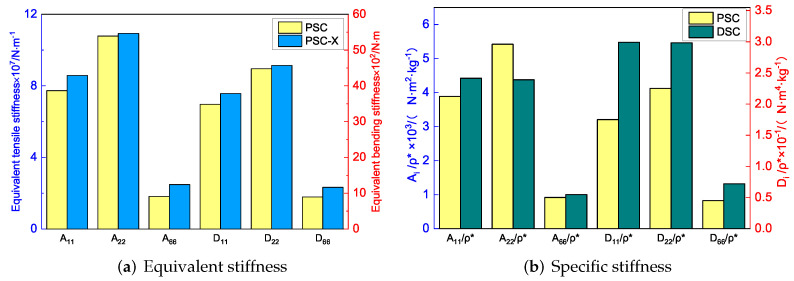
Comparison of equivalent stiffness and specific stiffness between sandwich panels with PSC-X, DSC and PSC.

To study the performance of the improved sandwich panels, the homogenization method was adopted to calculate the equivalent stiffness and equivalent density. Figure 18 shows the comparison of equivalent stiffness and specific stiffness between the improved panels and the original panel. It can be seen that the equivalent stiffness of the sandwich panel with PSC-X has been improved to a certain extent in all directions. Moreover, the NPR of the core layer increased from −0.585 to −0.725, which further resulted in the increase of shear modulus and shear resistance. The increase in NPR indicates the auxetic phenomenon was more significant, which was closely related to the deformation characteristics of the panel. The deformations of four corners in the cell core were limited by the added X-shaped ligaments. As a re-entrant structure, the NPR of sandwich panels with PSC-X mainly depended on the movement of triangles on both sides, which was not limited by the X-shaped ligaments.

To improve the specific stiffness, a sandwich panel with a double-arc star-shaped honeycomb (DSC) was proposed. Figure 18b compares the specific stiffness between SP-DSC and the original panel. Except for $A_{22}/\rho^{*}$, the specific stiffness of SP-DSC was greater than that of the original panel, and the NPR of the core layer decreased from −0.585 to −0.345, indicating that the energy absorption characteristics were reduced. This change was closely related to the core cell form. Because of the X-shaped ligaments, the connection between the SP-DSC parts was closer, and the deformation in the $x_{1}$ direction was more limited than in the original panel, resulting in a reduction in the NPR effect. In general, the specific stiffness of SP-DSC was better than the original panel, but the NPR effect was smaller.

Table 7 shows the recovered local stress, strain, and displacement fields at the midpoint of 2D-EPM for PSC, DSC, and PSC-X in case 2, respectively. The top facesheet was removed for a better view. It can be observed that the recovered stress and strain fields within the three core forms were quite different. The local stress and strain difference between the core layer and the facesheet in DSC was not as obvious as that of PSC and PSC-X, indicating that the core layer of DSC also played a more important role in the resist-bending. The bearing area of the facesheet was large in PSC due to the porosity in the core layer, resulting in the local stress and strain of the core layer being far less than that of the facesheet. The X-shaped ligaments were too weak to provide strong constraints on the facesheet in PSC-X, so its local stress and strain distributions were similar to those of PSC. The recovered local displacements showed that the local displacement in the middle of the core height was the largest, and the recovered maximum displacement was also consistent with the stiffness of the three plates.

**Figure 19:**
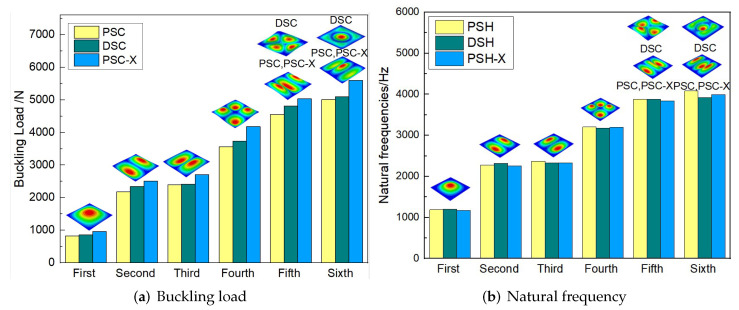
Comparison of buckling load and natural frequency of three panels.

Figure 19 compares the buckling and free vibration characteristics of three panels. Figure 19a shows that the buckling modes of the three panels were basically the same except for modes 5 and 6. The fifth and sixth buckling modes of sandwich panels with PSC and PSC-X were f(3,1) and f(1,3), respectively, while those of DSC were axisymmetric (f(m,n) was the mode shape with m,n denoting the half-waves along the $x_{1}$ and $x_{2}$ directions). The first six buckling loads of the sandwich panel with PSC-X were the largest, followed by SP-PSC and SP-DSC, which also reflected the relationship between the equivalent stiffness of the three panels with different core forms.

Figure 19b shows that the free vibration modes of the three panels were essentially the same except for modes 5 and 6. In modes 5 and 6, the mode shapes of PSC and PSC-X had three half-waves in $x_{1}$ and $x_{2}$ directions, respectively, while the mode shapes of DSC were centrosymmetric, which were related to the density gradient of different sandwich panels along $x_{1}$ and $x_{2}$ directions. In addition, the natural frequencies of the three panels were close to each other. The first three natural frequencies of the improved sandwich panel with PSC-X were the smallest, indicating its smaller specific stiffness. The natural frequency and NPR of SP-PSC were in the middle of three panels, indicating that the comprehensive performance of SP-PSC was better than two improved sandwich panels. One of the three panels could be selected for engineering application according to actual requirements.

## 7. Conclusions

In this article, the accuracy and efficiency of VAM-based 2D-EPM for sandwich panels with a petal star-triangular core (SP-PSC) were verified. The effects of geometric and material parameters of PSC (including angles $\theta_{1}$ and $\theta_{2}$ and height ratio) on the equivalent stiffness, buckling critical load, and natural frequency of sandwich panels were systematically investigated based on the 2D-EPM. The effective performance of the SP-PSC was compared with those of STC and SC to demonstrate its advantages. Then, two improved sandwich panels with PSC-X and DSC were proposed according to the stiffness characteristics of the panel, and their static and dynamic characteristics were compared with those of SP-PSC. The following conclusions can be drawn:

(1) The calculation efficiency of 2D-EPM for SP-PSC is about 20 times that of 3D-FEM, and the calculation accuracy can fully meet the engineering requirements. Except for $A_{22}$ and $D_{22}$, the equivalent stiffness and anti-buckling capacity decreased as the included angle $\theta_{1}$ between the adjacent petal walls increased. The change in equivalent stiffness was relatively insignificant with the increase in the included angle $\theta_{2}$ between adjacent concave inclined walls, as did the the corresponding anti-buckling capacity. The equivalent stiffness and the corresponding anti-buckling capacity decreased with the increase in height ratio. The change in natural frequency was affected by both the equivalent stiffness and the equivalent density.

(2) The fiber orientation had a significant effect on the NPR of the sandwich panel when the facesheet was made of unidirectional CFPR, and the change rule was as follows: the absolute value of Poisson’s ratio of SP-PSC increased with an increase in α when $\alpha <40^{\circ }$. The absolute value of Poisson’s ratio of SP-PSC decreased with an increase in α when $\alpha >50^{\circ }$, and the NPR effect was greatest when $\alpha =45^{\circ }$. The aforementioned rules held true when the material properties and geometric parameters $(\theta_{1},\theta_{2}$ and h/H) of the core layer were changed.

(3) Compared with STC and SC, SP-PSC had greater equivalent stiffness and better anti-buckling ability under the same conditions. The two improved panels improved the equivalent stiffness in different directions. The sandwich panel with PSC-X had higher stiffness and greater NPR effect with the same sizes as SP-PSC, so the corresponding buckling critical load had been improved. Compared with the SP-PSC, the specific stiffness, buckling critical loads, and local field distributions of the sandwich panel with DSC were improved. In the future, the energy absorption capacity of SP-PSC can be deeply studied, which lays the foundation for the practical application of this panel.

## Figures and Tables

**Table 1:** Comparison of first three buckling modes under different boundary conditions.

Modes	Models	Case 5 CCSS-Uniaxial	Case 6 CSFF-Uniaxial	Case 7 SSSS-Uniaxial
Mode 1	3D-FEM	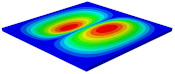 210.2 N	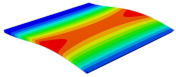 53.2 N	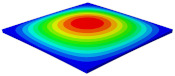 74.1 N
	2D-EPM	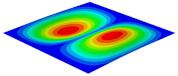 217.2 N (3.36%)	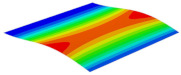 57.2 N (1.77%)	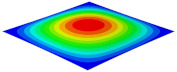 75.8 N (2.23%)
Mode 2	3D-FEM	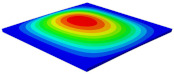 237.9 N	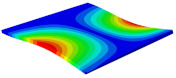 105.1 N	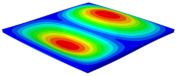 181.9 N
	2D-EPM	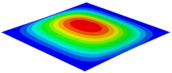 232.4 N (2.31%)	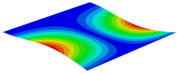 101.4 N (3.50%)	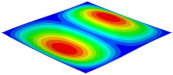 188.3 N (3.51%)
Mode 3	3D-FEM	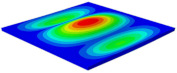 372.1 N	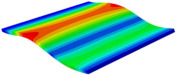 175.0 N	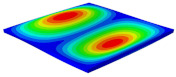 206.0 N
	2D-EPM	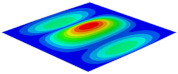 378.6 N (1.77%)	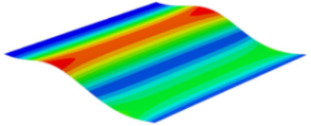 171.0 N (2.31%)	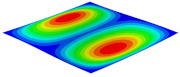 197.2 N (4.26%)
Mode 4	3D-FEM	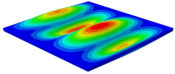 589.3 N	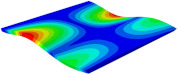 229.4 N	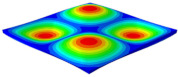 330.4 N
	2D-EPM	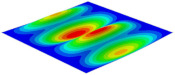 563.6 N (4.36%)	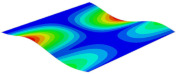 217.6 N (5.15%)	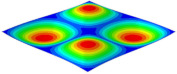 316.0 N (4.34%)

**Table 2:** Comparison of fist four natural frequencies predicted by two models under different BCs.

Orders	Case1 (CCCC)			Case2 (CCFF)			Case3 (SSSS)		
	3D-FEM	2D-EPM	Error	3D-FEM	2D-EPM	Error	3D-FEM	2D-EPM	Error
1	659.2	669.6	2.04%	408.4	417.3	2.16%	470.4	487.3	3.58%
2	1231.1	1307.0	6.14%	463.4	477.7	3.09%	907.0	953.4	5.12%
3	1297.4	1310.5	1.01%	783.0	763.6	2.49%	942.2	959.9	1.88%
4	1764.3	1857.1	5.26%	1062.6	1098.3	3.36%	1535.3	1500.0	2.30%

**Table 3:** Comparison of first eight natural frequencies of SP-PSC predicted by two models under CCCC BCs.

Modes	mode 1	mode 2	mode 3	mode 4
3D-FEM	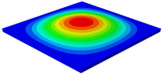 656.2 Hz	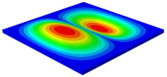 1231.4 Hz	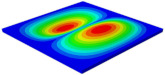 1297.4 Hz	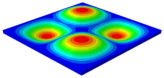 1764.2 Hz
2D-EPM	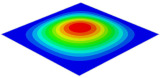 669.6 Hz (2.04%)	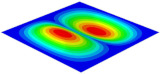 1307.0 Hz (6.14%)	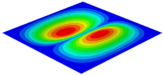 1131.0 Hz (1.01%)	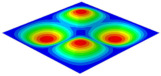 1857.1 Hz (5.26%)
Modes	mode 5	mode 6	mode 7	mode 8
3D-FEM	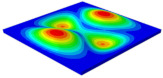 2243.7 Hz	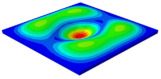 2294.6 Hz	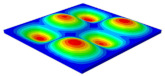 2753.4 Hz	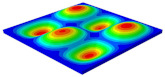 2817.7 Hz
2D-EPM	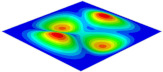 2215.9 Hz (1.24%)	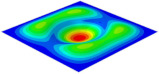 2235.3 Hz (1.99%)	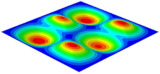 2689.8 Hz (2.31%)	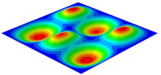 2694.9 Hz (4.36%)

**Table 4:** Comparison of calculation efficiency between two models.

Items	3D-FEM	2D-EPM	
		Unit Cell	2-D Plate
Element type	C3D20R	C3D20R	S4R
Number of elements	324,869	393,147	10,201
Number of nodes	160,332	75,682	10,000
Bending	96 min	/	5 min
Buckling	45 min	/	156 s
Vibration	27 min	/	121 s

**Table 5:** Comparison of recovered local stress, strain and displacement within the unit cell of PSC, STC and TH in case 2.

Type	PSC	STC	SC
σ	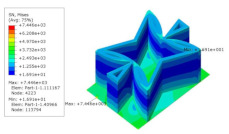 $\sigma_{\max}:7.446\times 10^{3}\;\mathrm{MPa}$	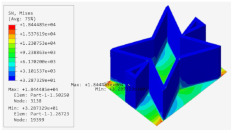 $\sigma_{\max}:1.881\times 10^{4}\;\mathrm{MPa}$	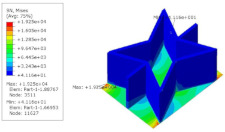 $\sigma_{\max}:1.925\times 10^{4}\;\mathrm{MPa}$
ε	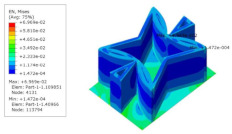 $\varepsilon_{\max}:6.969\times 10^{-2}$	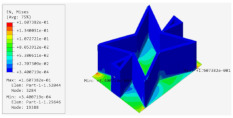 $\varepsilon_{\max}:1.641\times 10^{-1}$	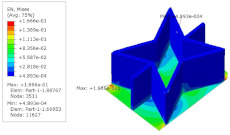 $\varepsilon_{\max}:1.666\times 10^{-1}$
*U*	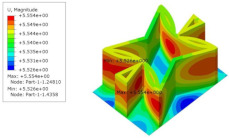 $U_{\max}:5.55\;$ mm	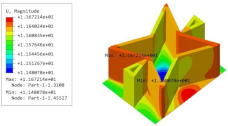 $U_{\max}:11.73\;$ mm	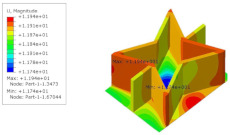 $U_{\max}:11.94\;$ mm

**Table 6:** Comparison of Poisson’s ratio of sandwich panels with PSC, STC and SC.

Types	PSC	STC	SC
Panel	$-2.1507469\times 10^{-2}$	$-9.7106579\times 10^{-3}$	$1.8372254\times 10^{-3}$
Core layer	$-5.8526654\times 10^{-1}$	$-4.8582582\times 10^{-1}$	$-4.7765997\times 10^{-1}$

**Table 7:** Comparison of recovered local stress, strain and displacement within the unit cell of PSC, DSC and PSC-X in case 2.

Type	PSC	PSC-X	DSC
σ	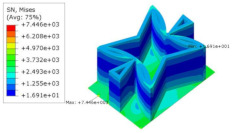 $\sigma_{\max}:7.446\times 10^{3}\;\mathrm{MPa}$	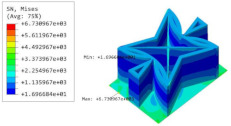 $\sigma_{\max}:6.731\times 10^{3}\;\mathrm{MPa}$	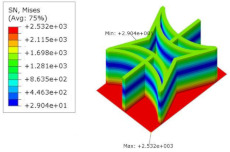 $\sigma_{\max}:2.532\times 10^{3}\;\mathrm{MPa}$
ϵ	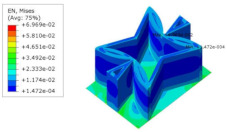 $\varepsilon_{\max}:6.969\times 10^{-2}$	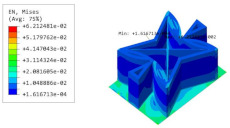 $\varepsilon_{\max}:6.212\times 10^{-2}$	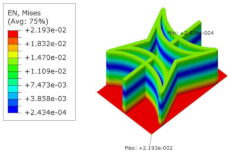 $\varepsilon_{\max}:2.193\times 10^{-2}$
*U*	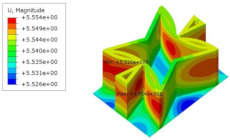 $U_{\max}:5.55\;$ mm	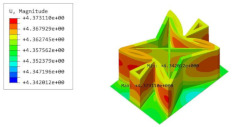 $U_{\max}:4.37\;$ mm	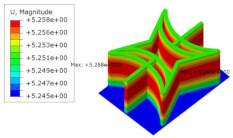 $U_{\max}:5.26\;$ mm

## Data Availability

Data available on request due to restrictions, e.g., privacy or ethical. The data presented in this study are available on request from the corresponding author. The data are not publicly available due to subsequent analyses and publications.
